# Event-Related Potential Evidence for Involuntary Consciousness During Implicit Memory Retrieval

**DOI:** 10.3389/fnbeh.2022.902175

**Published:** 2022-06-27

**Authors:** Xiu-Yuan Liang, Zi-Hao Guo, Xiao-Dong Wang, Xiao-Tao Guo, Jing-Wu Sun, Ming Wang, Hua-Wei Li, Lin Chen

**Affiliations:** ^1^Auditory Research Laboratory, School of Life Sciences, University of Science and Technology of China, Hefei, China; ^2^Faculty of Psychology, Southwest University, Chongqing, China; ^3^Department of Otorhinolaryngology-Head and Neck Surgery, The First Affiliated Hospital, University of Science and Technology of China, Hefei, China; ^4^Affiliated Eye and ENT Hospital of Fudan University, Shanghai, China

**Keywords:** implicit memory, consciousness, mismatch negativity, P3, pitch interval

## Abstract

Classical notion claims that a memory is implicit if has nothing to do with consciousness during the information retrieval from storage, or is otherwise explicit. Here, we demonstrate event-related potential evidence for involuntary consciousness during implicit memory retrieval. We designed a passive oddball paradigm for retrieval of implicit memory in which an auditory stream of Shepard tones with musical pitch interval contrasts were delivered to the subjects. These contrasts evoked a mismatch negativity response, which is an event-related potential and a neural marker of implicit memory, in the subjects with long-term musical training, but not in the subjects without. Notably, this response was followed by a salient P3 component which implies involvement of involuntary consciousness in the implicit memory retrieval. Finally, source analysis of the P3 revealed moving dipoles from the frontal lobe to the insula, a brain region closely related to conscious attention. Our study presents a case of involvement of involuntary consciousness in the implicit memory retrieval and suggests a potential challenge to the classical definition of implicit memory.

## Introduction

Numerous studies have been carried out for investigation into memory and learning since the 19th century. Classical notion claims that memory is implicit (non-declarative) if has nothing to do with consciousness during the information retrieval from storage, or is otherwise explicit (declarative). Under this notion, the so-called implicit memory refers to nonconscious memory abilities such as musical skills (e.g., play a piano). It is an important type of memory which often has an automatic quality and such a quality for the purpose of surviving in this complex diverse world is often innate. For instance, birds rely on it to fly in the sky and aquatic animals rely on it to live in the water. A well-known case for manifesting implicit memory is the amnesic patient H. M. who had undergone temporal lobe resection. H. M. preserved the memory related to motor skills and perceptual learning (Corkin, 1968; Moscovitch, 1995) as well as the types of memory that depended on the brain areas other than the medial temporal lobe, particularly on the hippocampus. H. M. also preserved the memory that did not depend on conscious awareness (Schacter, 1987; Squire, 1992). In addition, studies have revealed that selective injury to the medial temporal lobe leads to an isolated deficit in long-term memory (Scoville and Milner, 1957; Reber, 2013). The medial temporal lobe is equated with conscious-forms of memory and, therefore, with explicit memory (Degonda et al., 2005) which is commonly linked with the conscious awareness of memory retrieval.

Some studies show that an intact hippocampus is necessary for rapid associative learning with and without consciousness for long-term and short-term storage (Bennington and Polich, 1999; Henke, 2010). Imaging findings suggest that new semantic associations can be formed and retrieved by way of the medial temporal lobe without awareness of these associations (Henke et al., 2003a). The imaging studies further suggest that conceptual knowledge acquired during masking can be unconsciously retrieved (Henke et al., 2003b) and that implicit semantic associative learning engages the hippocampus and influences explicit memory (Degonda et al., 2005). Additionally, a visual electrophysiological study provides evidence for recognition lacking two hallmark explicit memory features: awareness of memory retrieval and facilitation by attentive encoding (Voss and Paller, 2009). All these studies suggest that consciousness seems to be a weak criterion for differentiating explicit and implicit memories. A new model has therefore been proposed in which memory systems are distinguished based on the processing characteristics involved rather than by the role of consciousness (Henke, 2010). The new model is an alternative to the classical memory model based on evidence from behavioral studies conducted in brain-impaired patients. To date, evidence for this new insight into distinguishing types of memory largely comes from subjective behavioral studies in brain lesion patients or neural imaging studies of explicit memory at spatial resolution. However, memory studies in healthy subjects at the temporal dimension during implicit memory retrieval is limited.

Mismatch negativity (MMN) is an auditory event-related potential (ERP) component and reflects the auditory cortical responses to novel stimuli (Näätänen et al., 1978, 2007; Partanen et al., 2013). MMN has been widely used as an effective neural marker for early auditory processing at a pre-attention stage (Luo et al., 2006; Gu et al., 2012; Wang et al., 2013; Guo et al., 2018). Importantly, MMN is regarded as a probe of implicit memory. P3 is another ERP component (Sutton et al., 1965) and has been claimed to be a neural marker of conscious perception by a number of investigators (Babiloni et al., 2006; Del Cul et al., 2007; Dehaene and Changeux, 2011; Rutiku et al., 2015). In the present study, we used MMN and P3 to investigate whether or not implicit memory is truly unassociated with consciousness. Specifically, we used an implicit memory paradigm (van Zuijen et al., 2006; Schröger, 2007) to expose a group of amateur musicians and a group of non-musicians to two different types of pitch intervals (e.g., one-pitch interval [C4 – C#4] and four-pitch interval [C4 – E4]). We found that a significant P3 component following the MMN was evoked in the amateur musicians, but was not in non-musicians. Our results provide ERP evidence that implicit memory retrieval of the musical pitch interval involves involuntary consciousness.

## Materials and Methods

### Whole-Head Electroencephalogram Recording

#### Participants

Thirty-six healthy students with normal hearing and no history of neurological disorders or learning abnormalities from the University of Science and Technology of China (USTC) participated in the present study (20 males, mean age = 21.85 years, SD = 1.84, right-handed; 16 females, mean age = 21.19 years, SD = 2.48, right-handed). Participants were allocated into the amateur musician group (10 males, mean age = 21.20, SD = 1.32; 8 females, mean age = 19.63, SD = 2.06) and the non-musician group (10 males, mean age = 22.50, SD = 2.12; 8 females, mean age = 22.75, SD = 1.83) according to their experience of musical training. Amateur musicians were recruited from Student Symphony Orchestra, Student Chinese Orchestra, and Student Choir at USTC and they had musical training more than 10 years for playing violin, piano, flute, Chinese zither, pipa, or singing. Amateur musicians and non-musicians were age- and sex-matched. The experimental protocols and procedures were reviewed and approved by the Biomedical Research Ethics Committee of the University of Science and Technology of China.

#### Stimuli

**FIGURE 1:**
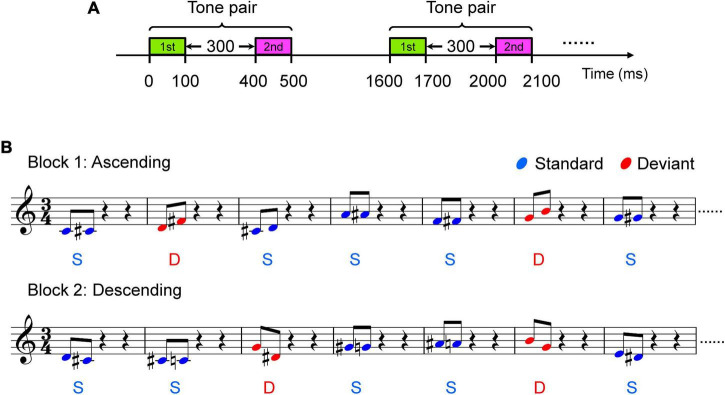
Two blocks of oddball paradigm. **(A)** Illustration of tone pairs. **(B)** Illustration of block 1 and block 2. Sample stave for two blocks is shown. Tones in block 1 are clockwise, which means pitch of the second tone of each tone pair is always rising. Tone pairs in block 1 with shorter distance (pitch interval = one semitone, e.g. C4 – #C4) are standard stimuli and those with a larger distance (pitch interval = four semitones, e.g. C4 – E4) are the deviant stimuli. Tones in block 2 are counterclockwise, which means pitch of the following tone of each tone pair is always falling. Tone pairs in block 2 with shorter distance (pitch interval = one semitone, e.g. #C4 – C4) are standard stimuli and those with a larger distance (pitch interval = four semitones, e.g. E4 – C4) are deviant stimuli.

Auditory stimuli used in the present study were Shepard tone pairs, which were synthesized with Praat software (Institute of Phonetic Sciences, University of Amsterdam, Netherlands^1^). The tone pairs were edited by Adobe Audition software. Each of the Shepard tones consists of many sinusoidal components locked at successive intervals of an octave simultaneously. In contrast to harmonic tones, which are well defined in terms of both pitch chroma and height, Shepard tones are well defined in terms of pitch class (C, C#, D, etc.) but poorly defined in terms of height, since the usual cues for height attribution are missing (Deutsch, 1986). The positions of the envelope within the lower octave peaked at C4 (262 Hz, *f*_min_ = 32.7 Hz), C#4 (277 Hz, *f*_min_ = 34.7 Hz), D4 (294 Hz, *f*_min_ = 36.8 Hz), D#4 (311 Hz, *f*_min_ = 39.0 Hz), E4 (330 Hz, *f*_min_ = 41.3 Hz), F4 (349 Hz, *f*_min_ = 43.8 Hz), F#4 (370 Hz, *f*_min_ = 46.4 Hz), G4 (392 Hz, *f*_min_ = 49.2 Hz), G#4 (415 Hz, *f*_min_ = 52.1 Hz), A4 (440 Hz, *f*_min_ = 55.2 Hz), A#4 (466 Hz, *f*_min_ = 58.5 Hz), and B4 (494 Hz, *f*_min_ = 62.0 Hz) (Table 1). Four types of Shepard tone pairs were presented: pairs with one-pitch and four-pitch clockwise intervals and one-pitch and four-pitch counterclockwise intervals (Table 2). Clockwise here means the second tone of each tone pair is always rising (ascending, Figure 1B), whereas counterclockwise means the second tone is always falling (descending, Figure 1B). The standard stimuli consisted of the one-pitch (clockwise) interval tone pairs, comprising C4 – C#4, C#4 – D4, D4 – D#4, D#4 – E4, F4 – F#4, G4 – G#4, A4 – A#4, and A#4 – B4, and the one-pitch (counterclockwise) interval tone pairs, including C#4 – C4, D4 – C#4, D#4 – D4, E4 – D#4, F#4 – F4, G#4 – G4, A#4 – A4, and B4 – A#4 (Table 2). The four-pitch (clockwise) interval tone pairs consisted of C4 – E4, C#4 – F4, D4 – F#4, D#4 – G4, E4 – G#4, F4 – A4, F#4 – A#4, and G4 – B4, and for the four-pitch (counterclockwise) interval tone pairs, the tones were reversed in direction, that is, E4 – C4, F4 – C#4, F#4 – D4, G4 – D#4, G#4 – E4, A4 – F4, A#4 – F#4, and B4 – G4 (Table 2). The one-pitch and four-pitch interval Shepard tone pairs served as the standard and deviant stimuli, respectively. Each tone was 100 ms in length, and each tone pair was 500 ms in length. The within-pair interval was 300 ms, stimulus onset asynchrony (i.e., from the onset of one tone-pair onset to the next) was set to 1,600 ms (Figure 1A).

**TABLE 1:** Minimum frequencies (in Hz) of Shepard tones.

Note name	Minimum Frequency (Hz)	Note name	Minimum Frequency (Hz)
C4	32.7	F#4	46.4
C#4	34.7	G4	49.2
D4	36.8	G#4	52.1
D#4	39.0	A4	55.2
E4	41.3	A#4	58.5
F4	43.8	B4	62.0

*C: do; D: re; E: mi; F: fa; G: so; A: la; B: xi.*

**TABLE 2:** Two blocks of tone pairs with one-pitch and four-pitch interval.

Clockwise (block 1)		Counterclockwise (block 2)	
One-pitch interval	Four-pitch intervals	One-pitch interval	Four-pitch intervals
C4 – C#4	C4 – E4	C#4 – C4	E4 – C4
C#4 – D4	C#4 – F4	D4 – C#4	F4 – C#4
D4 – D#4	D4 – F#4	D#4 – D4	F#4 – D4
D#4 – E4	D#4 – G4	E4 – D#4	G4 – D#4
F4 – F#4	E4 – G#4	F#4 – F4	G#4 – E4
G4 – G#4	F4 – A4	G#4 – G4	A4 – F4
A4 – A#4	F#4 – A#4	A#4 – A4	A#4 – F#4
A#4 – B4	G4 – B4	B4 – A#4	B4 – G4

#### Procedure

Participants sat in a comfortable sofa in an electrically shielded soundproof room. During whole-head electroencephalogram (EEG) recording, the subjects were instructed to ignore the auditory stimuli and watch a muted movie of their choice with subtitles. The stimuli were diotically presented through headphones (Sennheiser HD 25) at an intensity of ∼70 dB sound pressure level. To rule out the possibility that the MMN evoked by the deviant tone pairs was simply a response to an infrequent note in the deviant stimuli, we constructed the deviant tone pairs from the tones that also appeared among the standard stimuli (Table 2). The standard stimuli were presented with a probability of 7/8, and the deviant was presented with a probability of 1/8. In block 1 (Figure 1B, *upper panel* and Supplementary Audio 1), the stimuli were all the clockwise variants; each one-pitch interval tone pair was used as a standard stimulus with a probability of 7/64, and each four-pitch interval tone pair was used as a deviant stimulus with a probability of 1/64. In block 2 (Figure 1B, *lower panel* and Supplementary Audio 2), the stimuli were all the counterclockwise variants; each one-pitch interval tone pair was used as a standard stimulus with a probability of 7/64, and each four-pitch interval tone pair was used as a deviant stimulus with a probability of 1/64. The blocks were presented separately twice for 15 min each. Both amateur musician and non-musician participated in both blocks.

#### Electroencephalogram Recording and Preprocessing

Whole-head EEG signals were recorded using a SynAmps RT amplifier (NeuroScan, Charlotte, NC, United States) with a cap carrying 64 Ag/AgCl electrodes placed on the scalp at specific locations according to the extended international 10–20 system. Data were recorded at a sampling rate of 500 Hz. The reference electrode was attached to the tip of the nose, and electrode AFz served as the ground during the recording. To minimize the artifacts induced by eye-movement, horizontal and vertical eye movements were recorded using two bipolar electrooculography (EOG) electrodes. All electrode impedances were maintained below 5 kΩ. Preprocessing and data analysis were performed with NeuroScan and SPM12.^2^ Artifact rejection, filtering, and averaging were performed offline using Scan 4.3 (Neuroscan; Compumedics). The EEG data from the whole-head recordings were offline band pass (1–30 Hz) filtered with a finite impulse response filter. The filtered continuous data were then segmented into 900 ms epochs, including a 100 ms prestimulus baseline epoch. At the trial level, epochs with fluctuations in amplitude of at least 50 μV were considered artifacts and rejected expect for those of the EOG channels, which were excluded from the averaging. For illustration purposes, the ERPs of non-musicians and amateur musicians to the standard stimuli were averaged across the one-pitch interval tone-pair stimuli; a similar procedure was used for the responses to the deviant stimuli. The normality of the raw EEG data was assessed by using the Shapiro–Wilk (S-W) test. Paired-sample *t*-tests were performed at each sampling point throughout the epoch (−100 to 800 ms, one sampling point per 2 ms) for all subjects. Group-averaged deviant-minus-standard difference waveforms were then obtained by subtracting the ERPs evoked by the standard stimuli from those evoked by the deviant stimuli. The MMN component was then identified as a positive phase reversal over the mastoid processes (M1 and M2), and P3, was identified as the evoked signal immediately following the MMN component. Inspection of the grand-averaged difference wave suggested that the MMN peak amplitude was largest at FCz among the midline electrodes, which is consistent with findings in the literature indicating that the MMN component is prominent at the frontocentral sites (Näätänen et al., 2007). Two time windows were selected for amplitude measurements. Time window was 30-ms wide, ranging from 15 ms before the peak of the MMN recorded from electrode FCz to 15 ms after the peak. The other time window was 20-ms wide, ranging from 10 ms before the peak of the P3 component recorded from electrode FCz to 10 ms after the peak.

### Behavioral Test

To assess the relationship between the observed brain activities and the behavioral abilities after the EEG collection was finished, we described the rules of the two types of blocks to each subject and then performed a behavioral test using E-Prime software. We instructed each subject to press the button “1” when they determined the pitch interval of the tone pairs to be short (i.e., the one-pitch interval), and press the button “2” when they perceived the pitch interval of the tone pairs to be large (i.e., the four-pitch interval).

### Dipole Source Analysis

The localization of the dipoles generating the MMN and P3 activity was modeled using the BESA software package (Heuser-Link et al., 1992). The latency range where MMN responses were evident (510–560 ms) was selected for dipolar modeling. We first computed a 3D current source density (CSD) mapping with the grand average MMN. The CSD maps, expressed in μV/cm^2^, were constructed by calculating the volume current flow out of the brain through the skull into the skin by means of the surface Laplacian operator (second spatial derivative of the voltage distribution in tissue). This method reduces the effects of volume conduction to the scalp potential and allows for better visualization of the approximate locations of intracranial generators that contribute to MMN. We modeled the MMN response by a bilateral dipolar source and then conducted a local autoregressive average (LAURA) distributed linear inverse solution at the peak of global field power (GFP) of MMN waveform using a lead field (solution space) with the value of regularization of 0.03%. LAURA depicts the degree of CSD brain activity within derived source regions, which allows us to show the source of MMN located in the left and right auditory cortex. When it comes to P3 source localization, we selected the time interval of 560–660 ms and performed principal components analysis (PCA) to determine three pairs of symmetrical regional sources were required to model the grand average P3 (criterion: explained variance >1%). Then, the model developed on the grand average of all subjects was applied to the individual data and we conducted LAURA distributed linear inverse solution at the peak of GFP of P3 waveform using a lead field with the value of regularization of 0.03%.

### Dynamic Causal Modeling

Dynamic causal modeling (DCM) is an approach developed for connectivity analysis of functional magnetic resonance imaging (Friston et al., 2003). This method has been extended to magneto/encephalography (M/EEG) data (Garrido et al., 2007a; Boly et al., 2011). Most approaches to connectivity analysis of M/EEG data use functional connectivity measures, such as coherence, phase-synchronization or temporal correlations, which establish statistical dependencies between activities in two sources. Functional connectivity is useful, because it bases on the operational definition and is therefore independent of how the dependencies are caused (Garrido et al., 2007a). However, there are certain cases where causal interactions are the focus of interest. Here, DCM is particularly useful, because it uses the concept of effective connectivity. Effective connectivity refers explicitly to the influence one neuronal system exerts over another and can be estimated by perturbing the system and measuring the response by using Bayesian model inversion (Friston et al., 2003). In the context of EEG/MEG, DCM furnishes spatiotemporal, generative or forward models for evoked responses as measured with EEG/MEG (David et al., 2006; Kiebel et al., 2006). DCMs for MEG/EEG use neural mass models (David and Friston, 2003) to explain source activity in terms of the ensemble dynamics of interacting inhibitory and excitatory subpopulations of neurons, based on the model of Jansen and Rit (Jansen and Rit, 1995). Briefly, DCM provides an account of the interactions among cortical regions and allows one to make inferences about system parameters and investigate how these parameters are influenced by experimental factors; furthermore, by taking the marginal likelihood over the conditional density of the model parameters, one can estimate the probability of the data, given a particular model (Garrido et al., 2007b). This is known as the marginal likelihood or evidence and can be used to compare different models. Early components of the ERP have been linked to exogenous bottom-up stimulus-bound effects, whereas late components have been related to endogenous dynamics involving top-down influences (Garrido et al., 2007b).

#### Source Reconstruction and Model Specification

We applied 3D source reconstruction analysis for choosing the prior source locations in the following DCM model specification. Normalization parameters were obtained using unified segmentation of the subjects’ structural images (computerized tomography or T1 MRI) as implemented in the SPM software. Co-registration of electrode position and head model was performed for each subject prior to forward model computation. After the forward model was computed for each subject, the lead-field mapping of the cortical sources onto the measured signals was parameterized in terms of the location and orientation of each dipole source in the DCM (Garrido et al., 2008). Supplementary Table 1 and Supplementary Movie 1 show the coordinates for the locations of equivalent current dipoles (ECDs) in Montreal Neurology Institute (MNI) space (mm). The left and right primary auditory cortex (A1) were chosen as the cortical input stations for processing auditory information, both sides of temporal and frontal lobes were selected. By using these sources and prior knowledge about functional anatomy, we built a connectivity graph that featured an extrinsic input to the bilateral A1, which were connected to the corresponding ipsilateral temporal lobes, and both temporal lobes connected to the corresponding ipsilateral frontal lobes. Given this connectivity graph, specified in terms of its nodes and connections, we tested three models that differed in terms of the presence of reciprocal or recurrent connections: model F and model B had only forward and backward connections, respectively (Supplementary Figures 1A, B), while model FB had reciprocal connections, i.e., both forward and backward connections (Supplementary Figure 1C). In other words, model FB resembles recurrent dynamics or parallel bottom-up and top-down processing, whereas model F and model B emulate a simple bottom-up and top-down mechanism, respectively.

We selected a time window of interest spanning 490–560 ms to perform identical analyses for each subject of amateur musicians in the preprocessing stage of DCM. We modeled each active source, namely, each node in the network, with a single ECD in a conventional electromagnetic forward model. This electromagnetic model employed boundary element head models (Fuchs et al., 2001), with homogeneous and isotropic conductivity as an approximation to the brain, cerebrospinal fluid, skull, and scalp surfaces. Subject-specific head models were obtained using the inverse spatial normalization of a canonical mesh for each subject. Then, we used a two-stage approach in statistical analyses in this research, firstly, Bayesian model selection (BMS) was used to optimize the network architecture underlying electrophysiological responses to auditory stimulation in amateur musicians. Secondly, quantitative connectivity analysis was performed, conditioned upon the best model selected in Bayesian model comparison, searching for effective connectivity of the amateur musicians respond to auditory stimulation (Boly et al., 2011).

#### Bayesian Model Selection

The Bayesian brain hypothesis uses Bayesian probability theory to formulate perception as a constructive process based on internal or generative models, a free-energy principle has been proposed recently that accounts for action, perception and learning, the brain is an inference machine that actively predicts and explains its sensations. This generative model is decomposed into a likelihood (the probability of sensory data, given their causes) and a prior (the *a priori* probability of those causes) (Friston, 2010).

Bayesian model selection is used to decide which model, amongst a set of competing models, best explains the data (Penny et al., 2004). Inversion of a specific DCM involves optimizing a model (m) which provides two important quantities: the free-energy bound on the model-evidence *p*(*y*|*m*), used for model comparison, and the posterior or conditional density of the model parameters, *p*(θ|*y*,*m*). Specifically, DCM inversion corresponds to approximating the posterior probability of the parameters using variational Bayes (Friston et al., 2002). The aim is to minimize a free-energy bound on the log-evidence, with respect to a variational density, *q*(θ). When the free-energy is minimized; *q*(θ) = *p*(θ|*y*,*m*) and the free-energy *F* = −*ln*⁡*p*(*y*|*m*) approximates the negative marginal log-likelihood or negative log-evidence. After convergence, the variational density is used as an approximation to the desired conditional density and the log-evidence is used for model comparison. One often wants to compare different models and select the best before making statistical inferences on the basis of the conditional density. The best model, given the data, is the one with highest log-evidence *ln*⁡*p*(*y*|*m*) (assuming a uniform prior over models). Given two models m1 and m2 one can compare them by computing their Bayes factor, i.e., the difference in their log-evidences *ln*⁡*p*(*y*|*m*1)−*ln*⁡*p*(*y*|*m*2) (Garrido et al., 2007b).

In empirical or hierarchical Bayes models, the prior belief about the underlying causes of sensory input, *p*(θ), is optimized by higher hierarchical levels (i.e., higher-level brain areas) and provides top-down predictions on the most likely representations in lower levels. These “most likely” representations maximize the posterior belief or conditional density *p*(θ|*y*) of the causes of sensory data y. Bayes’ rule defines the conditional density as *p*(θ|*y*)∝*p*(θ)*p*(*y*|θ). This rule combines the top-down prior and a likelihood *p*(*y*|θ), which corresponds to the generative model used by the brain to predict its sensory input.

#### Quantitative Connectivity Analysis

We used the winning model (FB model, Figure 5A) from BMS above for final statistical analysis of the estimates of effective connectivity. In our DCMs, the effects of deviant stimuli (relative to standards) are modeled by scaling the effective connectivity in a trial-specific fashion. Although we tested for group differences in this (MMN-related) scaling, our primary interest was in differences in the underlying connection strengths mediated distributed responses to all stimuli. For analysis of quantitative connectivity, we compared the connectivity estimates (from the best model) by using paired sample *t*-tests, and then tested for differences in connection strength among the forward, backward, and lateral connections of the two hemispheres.

## Results

### A Robust Mismatch Negativity Response Was Evoked in Amateur Musicians but Not in Non-musicians

Participants were allocated into an “amateur musician” group and a “non-musician” group according to whether they had obtained long-term musical learning, such as playing an instrument. The groups did not differ in age or the proportion of sexes. To investigate implicit memory, we used a classical auditory oddball paradigm in which implicit memory can be probed with the evoked MMN.

**FIGURE 2:**
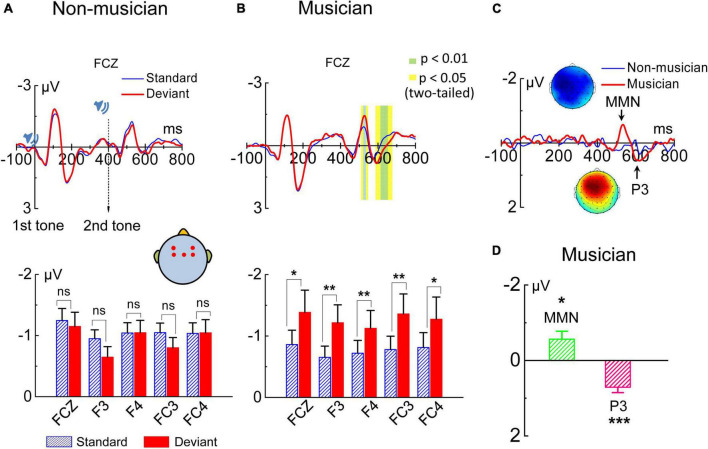
Grand average event-related potential (ERP) at electrode FCz. ERPs evoked by standard and deviant tone pairs, as well as deviant-minus-standard difference waveforms are plotted for groups of non-musicians and amateur musicians. **(A)** No MMN evoked in the non-musician group (*p* ≥ 0.05). **(B)** Amateur musician group extracted significant MMN amplitudes. Significant effects are marked with green and yellow areas in the waveforms. In the bar plots, significant effects are marked with asterisks (*p ≤ 0.05, **p ≤ 0.01). **(C)** Difference wave evoked in non-musicians (blue lines) and amateur musicians (red lines). ERP waveforms at the peak amplitude of MMN and P3 in the oddball paradigm of amateur musician group and the corresponding topographic map of MMN and P3 are shown. **(D)** Bar plots for MMN and P3 in the amateur musician group. Significant effects are marked with asterisks (*p ≤ 0.05, ***p ≤ 0.001).

The grand-averaged ERPs in response to the auditory stimuli were calculated with recordings from the FCz electrode for each subject. Paired sample *t*-tests were performed at each sampling point throughout the whole epoch (−100 to 800 ms, one sampling point per 2 ms) for each subject in the amateur musician group and in the non-musician group. Results obtained from amateur musicians showed that the ERP responses to the standard and deviant stimuli differed significantly (*p* < 0.05, two-tailed) at two time windows: 518–546 ms (i.e., 118–146 ms after the onset of the second tone in the tone pair), and 586–666 ms (i.e., 186–266 ms after the onset of the second tone) but did not differ significantly outside these time windows (Figure 2B). However, the EEG data of the non-musicians showed no significant difference (*p* > 0.05, two-tailed) between the ERP responses elicited by standard and deviant stimuli (Figure 2A). We next used the time window selected from 515 to 545 ms post stimulus onset, i.e., 115–145 ms after the onset of the second tone in the tone pair for MMN analysis. The MMN amplitudes, calculated from the recordings from two pairs of electrodes on the left (F3 and FC3) and right (F4 and FC4) sides of the scalp, revealed that MMN could be evoked from the changes in pitch interval in amateur musicians but not in non-musicians (Figures 2A,B). Different waves evoked by amateur musicians and non-musicians, as well as topographic map of MMN and P3 were shown in Figure 2C. Independent sample *t*-tests (two-tailed) showed that the amplitudes of the MMN component of the ERPs evoked by the amateur musicians were significant (Figure 2D). Given that this study was designed with a classically strict implicit memory retrieval paradigm, the MMN evoked by the changes in pitch interval evoked in the amateur musicians reflects their capacity for implicit memory retrieval.

To further investigate the MMN response, we analyzed the localization of the dipoles generating the MMN activity. The CSD mapping of MMN showed on the scalp surface a negative polarity over the frontocentral site and a positive polarity around the inferotemporal site (Figure 4A), indicating bilateral temporal generators accounting for MMN responses to tone pairs of four-pitch interval. Local autoregressive average (LAURA), a distributed source analysis, and dipole solution, a discrete source analysis, further confirmed that the generators of the MMN are located in the left and right temporal cortex in the musical group (Figure 4A). And the dipole strength of grand average MMN indicated a left hemisphere dominance of MMN in response to tone pairs of four-pitch interval, in line with the model in which left hemisphere being primary filling in the detailed pitch interval structure (Peretz, 1990; Warren, 2008).

### A Robust P3 Response Was Evoked in Amateur Musicians

Surprisingly, the whole-head EEG recordings showed that the amateur musicians evoked a significant P3 component followed the MMN. Paired sample *t*-tests performed at each sampling point throughout the epoch (−100 to 800 ms, one sampling point per 2 ms) for subjects revealed that the ERPs of the amateur musicians evoked by the standard and deviant stimuli differed significantly at 586–666 ms (i.e., 186–266 ms after the onset of the second tone of the tone pair). The grand averages of the ERP waveforms in response to the standard and deviant stimuli over the 610–630 ms time window for electrode FCz across non-musicians and amateur musicians are shown in Figures 2A,B. Independent samples *t*-tests (two-tailed) were further performed for each of the two types of stimuli, and the results showed there was a significant difference between the ERP amplitudes evoked by the standard and deviant stimuli of the amateur musicians (Figure 2B); no significant difference was shown in non-musicians. Therefore, we concluded that significant P3 could be elicited in response to changes in the pitch interval under the classical oddball paradigm in amateur musicians but not in non-musicians (Figures 2A,B). Independent sample *t*-tests (two-tailed) revealed that the amplitudes of the P3 component of the ERPs evoked by the amateur musicians were statistically significant (Figure 2D).

Next, source localization of the dipoles generating the P3 activity was analyzed. The CSD mapping of P3 showed on the scalp surface a positive polarity over the frontocentral site and a negative polarity around the inferotemporal site (Figure 4B), indicating bilateral generators accounting for P3 responses to the tone pairs of four-pitch interval. LAURA showed that the generators of the P3 are located in the frontal lobe and insula (Figure 4B). Importantly, 3D source movie revealed the dipoles moved from the frontal lobe (Figure 4B and Supplementary Movie 2), which has been associated with unconscious attention (Stuss and Alexander, 2000; Polich, 2007; Axelrod et al., 2015) to the insula (Figure 4B and Supplementary Movie 2), which is known to be highly dependent on conscious attention to stimuli according to previous studies (Bekinschtein et al., 2009; Citherlet et al., 2019). Moreover, P3 has often been claimed to be a key signature of conscious perception (Babiloni et al., 2006; Del Cul et al., 2007; Dehaene and Changeux, 2011; Salti et al., 2012; Rutiku et al., 2015).

### Amateur Musicians Showed Better Behavioral Performance Than Non-musicians

**FIGURE 3:**
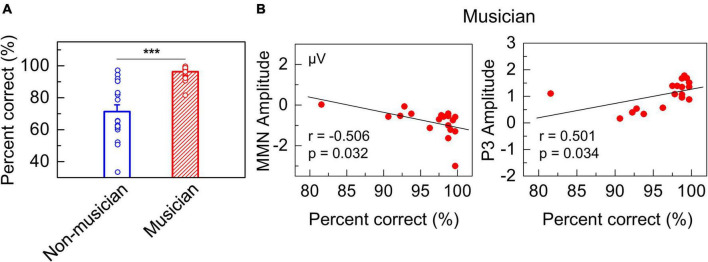
Behavioral results. **(A)** Plots showing significant difference in percent of correct responses between groups of amateur musicians and non-musicians (***p < 0.001). **(B)** The left panel shows significant correlations between the percent of correct responses and MMN amplitude in amateur musicians. The right panel shows significant correlations between the percent of correct responses and P3 amplitude.

According to behavioral tests, the performance of amateur musicians was significantly better than that of non-musicians. Responses to the behavioral test by amateur musicians and non-musicians were recorded by E-Prime software. We tested the percentage of correct responses of subjects in two groups by using independent sample *t*-tests (two-tailed). The results showed that the performance of the two groups was significantly different, in which the percentage of correct responses by the amateur musicians was much higher than that by the non-musicians (Figure 3A). Moreover, Pearson’s correlation analysis was performed to assess the correlation among the MMN, P3, and behavioral performance of the amateur musicians. The results showed that there was a significant correlation between ERP amplitudes and accuracy in the behavioral test (Figure 3B: *r* = −0.506, *p* = 0.032; Figure 3B: *r* = 0.501, *p* = 0.034).

**FIGURE 4:**
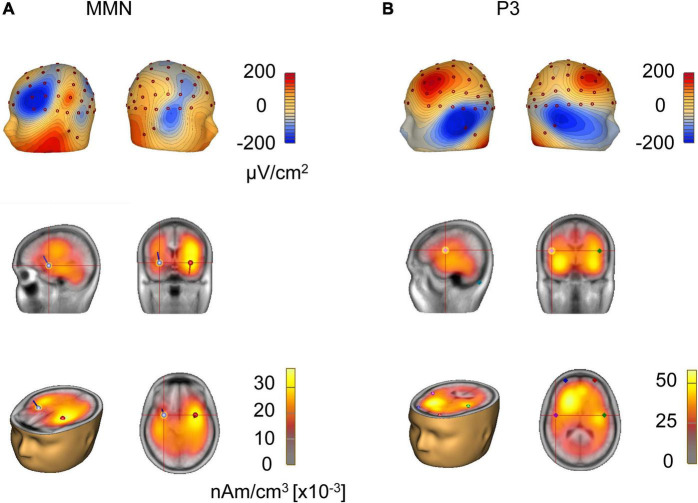
Source analysis of MMN and P3. **(A)** Current source density topography at the peak of global field power of grand-averaged MMN in response to tone pairs of four-pitch interval. Source localization estimated by local autoregressive average and dipole solution of MMN in response to tone pairs of four-pitch interval. **(B)** Current source density topography at the peak of global field power of grand-averaged P3 in response to tone pairs of four-pitch interval. Source localization estimated by local autoregressive average and dipole solution of P3 in response to tone pairs of four-pitch interval.

**FIGURE 5:**
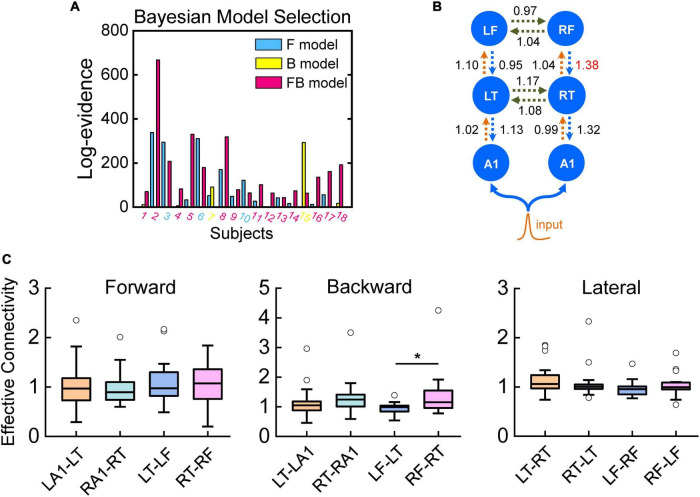
Quantitative effective connectivity. Bayesian model selection among DCMs for the three models, F, B, and FB, expressed relative to a DCM in which no connections were allowed to change (null model). The graphs show the free energy approximation to the log-evidence. **(A)** Log-evidence for models F, B, and FB for each amateur musician (relative to the null model). The diamond attributed to each subject identifies the best model on the basis of the subject’s highest log-evidence. Log-evidence at the group level, i.e., pooled over subjects, for the three models. **(B)** Effective connectivity of the FB model. **(C)** In the bar plots, significant difference on the backward connection from the frontal lobe to the temporal lobe of the right hemisphere compared to the backward connection from frontal lobe to temporal lobe of the left hemisphere and no difference between the two hemispheres on the forward and lateral connections.

### Effective Connectivity From the Right Frontal Lobe to the Ipsilateral Temporal Lobe in Amateur Musicians

To reveal the detailed processing characteristics of implicit memory retrieval, we used DCM of the ERPs to quantify effective connectivity of the amateur musicians. Three models, differed in the areas and connections involved (Figure 5), were constructed and BMS was used to compare these three models. Fixed effects family level analysis showed that models including two frontal sources with both forward and backward connections could best explain the ERP responses in amateur musicians (Figure 5A). Then, we used the winning model, i.e., FB model, from BMS for the final statistical analysis of the calculation of effective connectivity. To further analyze the quantitative connectivity and compare the memory process between the two hemispheres, we analyzed the connectivity calculates from the best model using paired sample *t*-tests. The data indicated a significant difference on the backward connection from the frontal lobe to the temporal lobe of the right hemisphere compared to the backward connection from the frontal lobe to the temporal lobe of the left hemisphere (Figures 5B,C) and no difference between the two hemispheres on the forward or lateral connections (Figures 5B,C).

## Discussion

The present study was carried out in healthy subjects at the temporal dimension during implicit memory retrieval. Our results demonstrate a robust P3 component during implicit memory retrieval of musical pitch interval in musicians, which is believed by many to be an indicator of involuntary consciousness accompanying the implicit memory retrieval. Specifically, our results suggest that implicit and explicit memories may not necessarily have to be clearly differentiated by whether consciousness is involved and that aspect of memory processing, such as top-down process, might be considered as an effective factor in defining types of memory. Our study raises the possibility that consciousness, to some extent, may not be associated with the definition of memory. In our study, EEG was recorded under a traditional oddball paradigm to directly compare the auditory processing of different musical pitch intervals in amateur musicians and non-musicians. We provided a behavioral test to the two groups of participants and assessed their performance in terms of the percentage correct responses. The electrophysiological results showed that large MMN and P3 amplitudes could be elicited in subjects with long-term musical learning but not in participants without musical training for either stimulus block (Figures 2A,B). Furthermore, the amateur musicians behaviorally outperformed non-musicians (Figure 3A), which is associated with the MMN component evoked by the oddball paradigm among the amateur musicians. The behavioral findings demonstrate a significant correlation with EEG results, i.e., larger amplitudes were correlated with higher accuracy in the behavioral test (Figure 3B). These results are consistent with previous studies in which amateur musicians performed better than non-musicians when detecting speech in noise and demonstrate enhanced subcortical auditory and audiovisual encoding of speech and music sounds (Musacchia et al., 2007; Song et al., 2012). Additionally, brain electrical source analysis of the P3 component evoked by amateur musicians revealed that the dipoles moved from the frontal lobe to the insula (Figure 4B and Supplementary Movie 2), which is known to be highly dependent on conscious attention to stimuli according to previous studies (Bekinschtein et al., 2009; Citherlet et al., 2019). Notably, analysis of the P3 component suggests that the implicit memory retrieval of musical pitch intervals in this study may involve unconscious access. The effective connectivity obtained by DCM analysis also reveals a significant increase in backward connectivity, namely, top-down processing from the right frontal lobe to the ipsilateral temporal lobe, in amateur musicians (Figures 5B,C). This is in line with some evidence from auditory and visual studies of humans and animals supporting that explicit memory retrieval is under the active executive control of top-down processes from the prefrontal cortex (Hasegawa et al., 1998; Tomita et al., 1999; Miyashita, 2004; Kostopoulos and Petrides, 2016; Risius et al., 2019). This suggests that implicit and explicit memories may share a similar underlying neurocognitive mechanism. Altogether, our study presents a case of involvement of involuntary consciousness during implicit memory retrieval and suggests a potential challenge to the classical definition of implicit memory.

### Implicit Memory Retrieval of Musical Pitch Interval May Involve the Brain Circuit Associated With Involuntary Consciousness

Traditionally, when we refer to implicit memory, we mean memory defined as obtained knowledge that is not available to conscious access (Schacter and Graf, 1986). For instance, learning to ride a bicycle initially involves conscious attention to one’s body and the bicycle. Later, riding eventually becomes an automatic activity, which can be regarded as implicit memory shaped through learning and does not necessarily involve awareness of the memory (Kandel, 2006). Analogous to the implicit memory of riding a bicycle, amateur musicians who undergo long-term musical learning is a good model for studying implicit memory. In our study, significant MMN responses can be elicited in amateur musicians (Figure 2B), which has been widely used as an effective electrophysiological signature for studies of early auditory processing (Luo et al., 2006; Gu et al., 2012; Wang et al., 2013; Guo et al., 2018). It is also regarded by many as a probe of implicit memory. In our experiment, the evocation of MMN by amateur musicians in the preattentive stage suggests that long-term musical learning promotes the generation of implicit musical memory in their memory storage systems. Surprisingly, our neurophysiological measures reveal a significant P3 amplitude following the MMN in amateur musicians (Figure 2B). P3 is a positive-going component of evoked-potential waveforms that has been associated with the processing of unexpected events and was first reported in 1965 (Sutton et al., 1965). P3 is elicited most commonly in the context of the auditory oddball paradigm, where it can be used as an index of the involuntary shift of attentional resources toward novel stimuli and can be evoked under attending or ignoring situations (Ritter et al., 1968; Kok, 2001; Horváth et al., 2008). Some studies have proposed that MMN, especially frontal MMN, is associated with P3, indicating involuntary attention switching (Schröger, 1996; Rinne et al., 2000). Because our results show a salient P3 component evoked among the amateur musicians under the oddball paradigm for the retrieval of implicit memory, we propose that the attention of amateur musicians was drawn involuntarily by the deviant stimuli. Non-musicians apparently could not distinguish the rare stimuli, as suggested by the absence of P3 component, which depends on the ability to process task-relevant stimuli and reflects event classification *via* the correlation of attention and the working memory network (Kok, 2001). In addition, source localization of the dipoles generating the activity of the P3 component modeled by the BESA software package reveals the dipoles moving from the frontal lobe (Figure 4B and Supplementary Movie 2), suggesting an unconscious attention (Stuss and Alexander, 2000; Polich, 2007; Axelrod et al., 2015) is associated with the insula (Figure 4B and Supplementary Video 1), which is known to be highly dependent on conscious attention to stimuli (Bekinschtein et al., 2009; Citherlet et al., 2019). Thus, we speculate that there is a transition from unconsciousness to consciousness during the P3 component of implicit memory and that the implicit memory retrieval of musical pitch interval may involve the consciousness. Accumulating evidence from studies on explicit memory indicates that an intact hippocampus is necessary for rapid associative learning with and without consciousness (Henke, 2010). In this vein, our study on implicit memory retrieval provides ERP evidence supporting the view that consciousness may be an inadequate criterion for differentiating types of memory.

### Implicit Memory and Explicit Memory May Share an Analogous Underlying Neurocognitive Characteristic: Top-Down Processing

Top-down regulation is an experience-dependent process that originates from the prefrontal cortex, carrying an abundant amount of prior knowledge and transmitting information synthesized from experience that facilitates an individual’s interpretation of input information (Tomita et al., 1999; Lee and D’Esposito, 2012). Moreover, such stored information provides context and meaning to sensory inputs, which is central to high-level cognition of basic auditory processing and visual recognition (Sohoglu et al., 2012; Gilbert and Li, 2013). A previous study in speech perception suggests that a top-down mechanism would be reflected with abstract computations in the inferior frontal gyrus (IFG) being modulated before sensory-related processing in the superior temporal gyrus (STG) (Sohoglu et al., 2012). Behavioral evidence from recent studies have shown that musical learning also has a close relationship with top-down pathway and suggest that top-down regulation is involved in the formation of music-related memory in the auditory processing (Kraus and Chandrasekaran, 2010; Strait et al., 2010). Since explicit memory is characterized by knowledge that involves conscious recollection, recall, or recognition (Ettlinger et al., 2011), evidence from the auditory and visual studies of humans and animals supports the notion that explicit memory retrieval is under the executive active control of top-down processes. Many studies on memory research have reported that episodic memory, which is classified as explicit memory, is associated with conscious encoding (Rombouts et al., 1997; Henke et al., 1999; Staresina and Davachi, 2009) but may not involve consciousness (Henke et al., 2003a; Degonda et al., 2005). Therefore, one model proposes that different types of memory are distinguished according to the processing operations involved rather than by consciousness (Henke, 2010). However, to the best of our knowledge, the majority of previous studies on memory processing are based on explicit memory research (Tomita et al., 1999; Boly et al., 2011), little work has been done from the perspective of implicit memory retrieval to examine whether implicit memory and explicit memory can be distinguished based on consciousness. In the present study, measures of effective connectivity show distinguishable backward connectivity from the right frontal lobe to the ipsilateral temporal lobe in amateur musicians (Figure 5), which demonstrates that top-down processing is involved in the implicit memory retrieval of musical pitch intervals. These findings are consistent with those indicating the involvement of top-down processing in the retrieval of explicit memory (Hasegawa et al., 1998; Tomita et al., 1999; Miyashita, 2004). Combining the above points, we further suggest that, to some extent, implicit and explicit memory may share an analogous underlying top-down neurocognitive mechanism. Indeed, all of these results clearly demonstrate that long-term musical learning induces brain plasticity, which accounts for the activation of top-down processing (i.e., backward frontotemporal connectivity). Additionally, in the present study, the better behavioral performance (Figure 3A) in the selection of pitch interval, as well as the more robust MMN response elicited in amateur musicians than in non-musicians, suggests that amateur musicians have prior knowledge of the pitch interval and that musical training can induce their abilities to detect the pitch interval of a tone pair *via* top-down processing. Therefore, we suggest that top-down signals and prior knowledge-related and higher-order recognition processing participate in the retrieval of the implicit memory of pitch interval.

### Bayesian Inference and Predictive Coding in the Brain: The Mechanism Underlying the Automatic Detection of Changes

Bayesian inference has been proposed as a basic principle for brain function, which is based on an internal generative model used by the brain to predict sensory input, that comprises a distribution over sensory data given an external cause (the sensory data likelihood) and a prior distribution over different causes (Summerfield et al., 2006; Joos et al., 2014). The predictive coding framework is a well-known hypothesis of the mechanism of human sensory perception. The central assumption in predictive coding theory is that the activity in the nervous system reflects a process of matching internally generated predictions, which anticipates the forthcoming sensory environment, to external stimulation (Rao and Ballard, 1999; Heekeren et al., 2008; Rauss and Pourtois, 2013). Predictive coding, under which the brain is regarded as a hierarchically organized cortical system, is a general theory of perceptual inference, and recently, it has been proposed as the mechanism underlying the generation of the MMN component and has been formulated in terms of empirical Bayesian models of perceptual learning and inference (Näätänen et al., 2007; Garrido et al., 2009). As we mentioned above, the MMN component has been identified as a typical neurobiological marker for error (uncertainty or unexpectedness) detection caused by deviant inputs. Previous studies have also shown that recurrent dynamics generate evoked brain responses in cortical networks, and feed-forward connectivity is sufficient to generate early ERP components; conversely, late components are mediated by backward connections (Garrido et al., 2007a; Boly et al., 2011). *Via* the results obtained from BMS, we found that the best model includes modulations of both forward and backward connections (FB model, Figure 5A). Our results support and extend findings showing that a frontotemporal network is involved in generating mismatch responses and that this generation entails an interaction between top-down and bottom-up exchanges between cortical sources, in line with the results from other studies (Kiebel et al., 2009).

Next, the Bayesian brain model proposes that our brain works in a Bayesian way under the free-energy principle, which asserts that any adaptive change made by a biological system or organism must minimize its free energy (i.e., reduce environmental uncertainty, unexpected or unpredicted sensations) (Edwards et al., 2012). In this model, the Bayesian brain can be conceptualized as a probability machine that always makes predictions about the world and updates them based on what it senses (Friston, 2010). Thus, we suggest that the brains of amateur musicians can use prior knowledge (implicit memory of musical pitch interval) to predict the incoming sensory inputs to reduce the uncertainty of the environment (i.e., prediction error). Neuronal activity reflects attempts to minimize or reduce prediction error (i.e., uncertainty) to estimate the most likely cause of the input and represent the states of the world according to the free-energy principle. Repeated stimuli reduce the prediction error from bottom-up regulation, while the detection of deviant stimuli may lead to the increase of the prediction error. In the current study, we propose that due to long-term musical learning, amateur musicians possess the implicit memory related to musical elements. Such participants can not only determine that the stimuli are being presented in pairs but also recognize the inner rule of the stimuli, i.e., pitch interval. In this circumstance, the predictive top-down signals from the frontal lobe to the temporal lobe associated with two characteristics of the stimuli (i.e., tone pairing and pitch interval) were expected by the prediction units of the amateur musicians according to information about the stimuli they had previously acquired. If the predicted stimulus is consistent with the incoming stimulus, the prediction error will gradually be minimized based on the free-energy principle. Otherwise, when a deviant stimulus with a pitch interval different from that of the predicted stimulus is presented, after the real stimuli heard from the headphone are compared to the sounds predicted by the amateur musician’s prediction error units, the prediction error will increase, which will result in changes in the amplitude, direction and position of the dipole in the musician’s brain. Then, the appearance of the MMN component of the ERP, a marker of automatic error detection, will be elicited because of the failure to minimize surprise, which leads to an increase in entropy in the brain system; and the P3 component of the ERP, an index of involuntary attention switch, is elicited. While non-musicians can only perceive the paired tone rule, they are unable to distinguish the difference in the pitch interval between the standard and incoming deviant stimuli, and their prediction error will be unchanged. Thus, an MMN cannot be elicited by the deviant stimuli in non-musicians in the present paradigm.

### Amateur Musician Is a Good Model for Studying Implicit Memory

As we all know, music can move us, and musical learning plays a significant role in various respects of human hearing skills as well as different ages. For instance, in terms of infancy, active music classes in infancy enhance musical, communicative, and social development (Gerry et al., 2012). Studies of children showed that musical learning during early childhood improves the neural encoding of speech in noise; besides, speech segmentation, pre-attentive processing of syllabic duration are directly facilitated by musical training (Chobert et al., 2012; Strait et al., 2012; Virtala et al., 2012; Francois et al., 2013). Moreover, adult research has examined brainstem encoding of linguistic pitch and that musicians show more robust and faithful encoding compared with non-musicians (Wong et al., 2007). Not only functional advantages but also structural changes have occurred in the brain of musicians, such as enlarged gray and white matter, and better developed cognitive function of left temporal correlated with verbal memory in musicians (Chan et al., 1998; Gaser and Schlaug, 2003; Chobert et al., 2011; Zatorre et al., 2012). Interestingly, we can hardly be surprised, meanwhile, that music lessons improve children’s IQ (Schellenberg and Hallam, 2005), given that they will nourish general faculties such as memory, coordination, and attentiveness that they will nourish general faculties such as memory, coordination as well as attentiveness. Additionally, music skills have also been found to correlate significantly with both phonological awareness and reading development (Anvari et al., 2002).

Shepard tone used in the current research was generated by Shepard in 1964; each tone consisted of many sinusoidal components locked at successive intervals of an octave and sounded simultaneous. We used this type of stimulus to enhance the challenge for participants in the processing of different pitch intervals. In our study, EEG results showed that the larger pitch intervals, namely, deviant stimuli, can elicit significant MMN responses in amateur musicians, which is a component of ERP indicating automatic change detection (Näätänen et al., 2007). However, no MMN responses could be evoked by participants without long-term musical learning. We can therefore propose that long-term musical learning induces adult’s automatic ability of processing pitch interval, and such capacity can be integrated into the existent automatic abilities. Consistent with previous studies, amateur musicians perform better than non-musicians when detecting speech in noise environment, as well as enhanced subcortical auditory and audiovisual encoding of speech and music sounds (Musacchia et al., 2007; Song et al., 2012). Additionally, previous research, for instance, the auditory brainstem responses when listening to musical intervals, has demonstrated results consistent with our study (Lee et al., 2009). Apart from this, the pianists also show increased neural activity (measured by magnetic source imaging) in the auditory cortex in response to hearing piano notes (Pantev et al., 1998; Brunelliere et al., 2009). Thus, we propose that long-term musical learning induces an adult’s ability of automatic pitch interval processing, and such capacity can be integrated into the automatic detection of implicit memory.

### Neural Correlate of Consciousness

Implicit memory retrieval of musical pitch interval in the current study seems to be highly related to the access neural correlate of consciousness. Ned Block describes how access neural correlate of consciousness (NCC) are distinct from phenomenal NCC: access conscious content is information about which is “broadcast” in the global workspace, while phenomenally conscious content is as different experience of red and green (Block, 2005). In other words, access conscious contents information about which is made available to the “consumer” systems of the brain: such as memory system, voluntary direction of attention system, perceptual categorization system, and more generally, system of rational control of action (Block, 2005). Two theories about neural basis of consciousness are put forward. One theory is Recurrent Processing Theory (RPT) (Lamme and Roelfsema, 2000; Lamme, 2006, 2010) and the other one is Global Neuronal Workspace Theory (GNWT) (Dehaene et al., 2006; Dehaene and Changeux, 2011). According to RPT, all perceptual organization required for vision of consciousness is achieved by the visual cortex and the frontal cortex has only modulatory influence to some extent. According to the GNWT, however, the dorsolateral prefrontal cortex (DLPFC) plays a critical role in mediating the conscious contents, at least in conscious “access” to the content information (Northoff and Lamme, 2020). In addition, GNWT with its focus on access rather than phenomenal consciousness points at the later brain activity (P300, more specifically P3b as observed in the present study) is regarded as the key signature of “global ignition,” which becomes available of sensory information for other brain areas, and access to consciousness (Sergent et al., 2005; Dehaene and Changeux, 2011). There is no unchallenged best hypothesis on P3b (Verleger, 2020) and more studies on different hypotheses needs to be tested against each other. The relationship between P3 and consciousness, as the case in our present study, requires more investigation in future study.

## Data Availability Statement

The original contributions presented in the study are included in the article/Supplementary Material, further inquiries can be directed to the corresponding author.

## Ethics Statement

The studies involving human participants were reviewed and approved by the Biomedical Research Ethics Committee of the University of Science and Technology of China. The participants provided their written informed consent to participate in this study.

## Author Contributions

LC and X-YL designed the research. X-YL and X-TG performed the research. X-YL, Z-HG, and X-DW analyzed the data. LC, X-YL, H-WL, J-WS, and MW wrote the manuscript. All authors contributed to the article and approved the submitted version.

## Conflict of Interest

The authors declare that the research was conducted in the absence of any commercial or financial relationships that could be construed as a potential conflict of interest.

## Publisher’s Note

All claims expressed in this article are solely those of the authors and do not necessarily represent those of their affiliated organizations, or those of the publisher, the editors and the reviewers. Any product that may be evaluated in this article, or claim that may be made by its manufacturer, is not guaranteed or endorsed by the publisher.

## Footnotes

1
www.praat.org

2
http://www.fil.ion.ucl.ac.uk/spm/
