# Supplementary material for: Event-Related Potential Evidence for Involuntary Consciousness During Implicit Memory Retrieval
Source: Front Behav Neurosci. 2022 Jun 27;16:902175. doi: 10.3389/fnbeh.2022.902175 (PMC9272755; doi:10.3389/fnbeh.2022.902175)
Supplement: Supplementary file 1 [file Image_1.pdf]

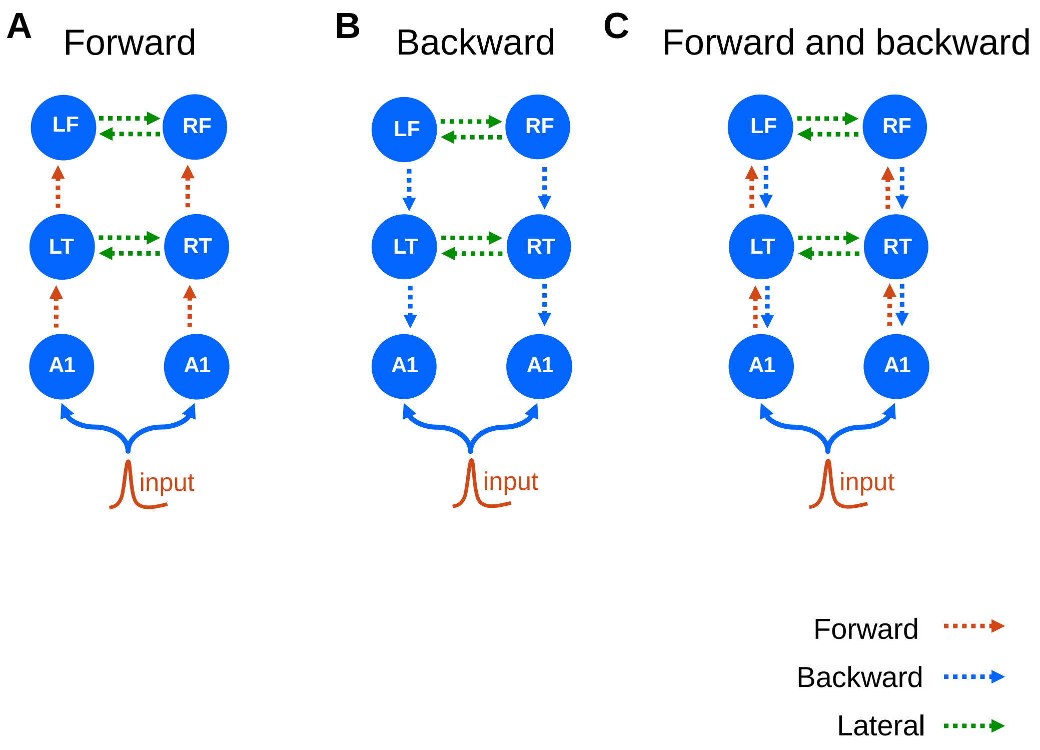

Supplementary Figure 1. Model specification. The sources comprising the network are connected with lateral (green), backward (blue) and/or forward (orange) connections as shown. A1, primary auditory cortex; LT, left temporal lobe; RT, right temporal lobe; LF, left frontal lobe; RF, right frontal lobe. Three different models were tested within the same architecture, with forward, with backward, and with forward and backward connections [**(A)**, **(B)**, and **(C)**, respectively].
