# Supplementary material for: Event-Related Potential Evidence for Involuntary Consciousness During Implicit Memory Retrieval
Source: Front Behav Neurosci. 2022 Jun 27;16:902175. doi: 10.3389/fnbeh.2022.902175 (PMC9272755; doi:10.3389/fnbeh.2022.902175)
Supplement: Supplementary file 2 [file Table_1.doc]

**Supplementary Table 1. Coordinates for locations of the ECDs in Montreal Neurology Institute space [mm]**

| Left primary auditory cortex | -42, -22, 7 |
| --- | --- |
| Right primary auditory cortex | 46, -14, 8 |
| Left temporal lobe | -47, 35, 2 |
| Right temporal lobe | 50, 35, 3 |
| Left frontal lobe | -24, 62, -3 |
| Right frontal lobe | 20, 61, -3 |
